# Policy experimentation: core concepts, political dynamics, governance and impacts

**DOI:** 10.1007/s11077-018-9321-9

**Published:** 2018-05-24

**Authors:** Dave Huitema, Andrew Jordan, Stefania Munaretto, Mikael Hildén

**Affiliations:** 1 Institute for Environmental Studies (IVM), Vrije Universiteit Amsterdam, De Boelelaan 1087, 1081 HV Amsterdam, The Netherlands; 2 Faculty of Management, Science, and Technology, Netherlands Open University, Valkenburgerweg 177, 6419 AT Heerlen, The Netherlands; 3 Tyndall Centre for Climate Change Research, School of Environmental Sciences, University of East Anglia, Norwich, NR4 7TJ UK; 4 Climate Change Program, Finnish Environment Institute, P.O. Box 140, 00260 Helsinki, Finland

## The experimentalist ‘turn’ in science

In the last two decades, many areas of the social sciences have embraced an ‘experimentalist turn’. It is well known for instance that experiments are a key ingredient in the emergence of behavioral economics, but they are also increasingly popular in sociology, political science, planning, and in architecture (see McDermott 2002). It seems that the potential advantages of experiments are better appreciated today than they were in the past.

But the turn towards experimentalism is not without its critics. In her passionate plea for more experimentation in *political science* for instance, McDermott (2002: 42) observes how many political scientists are hesitant: they are more interested in large-scale multiple regression work, lack training in experimentation, do not see how experiments could fit into a broader research strategy, and alternative movements in political science (such as constructivists and postmodernists) consider that experimental work is not able to capture complexities and nuances. Representing some of these criticisms, Howe (2004) suggests that experimentation is being oversold and highlights various complications, especially the trade-offs that exist between internal and external validity, the fact that causal inferences can be generated using many other research methods, and the difficulty of comparing governance interventions to new medications in medicine.

Even if McDermott (2002) and Howe completely disagree on the potential of experiments, they do agree that experiments should mainly be seen as a *research method.* But this is clearly not the only way in which experiments are viewed in practice; indeed there are many signs that the experimentalist turn is restricted to issues of methodology. Far from it. In an influential overview of the experimentalist turn in a range of disciplines, Ansell and Bartenberger (2016) suggest that the term experiment denotes a range of forms and activities. It is, for example, being used to refer to the design and evaluation of institutional arrangements, to the encouragement of social and political learning, and to the triggering of innovations and transitions (ibid.: 64). In other words, experimentalism is being actively equated with a distinct *approach to governing,* including but not limited to public policy.

This change in conceptualization is one reason why it could be interesting, also for policy scientists, to engage with the experimentalist turn, and this is exactly the purpose of this special issue. Our main goal is to identify the scope for mutual learning, between this turn and the policy sciences. What new insights, for example, does the new work on experimentation offer to those in the policy sciences who have been wrestling with experimentation for many years, even decades? And just as importantly, what can policy scientists add to the new experimentalist literature with respect to core concepts, methods, theoretical insights and empirical findings?

In this introductory paper, we seek to address these questions by exploring *experimentation in relation to climate governance.* This provides a good opportunity to showcase new experimentalist turn in action, in its various shapes and forms. Why this particular case? There is probably no field where the impact of the experimentalist turn has been greater than that of sustainability studies, of which the discussion on climate governance is but one part. At least two leading current approaches—transitions management (see for instance Voß et al. 2009; Berkhout et al. 2010) and adaptive co-management (see for instance Huitema et al. 2009)—regard experimentation as being foundational. Both treat experiments as the starting point (or seed) for desirable societal transformations (for an overview, see Den Uyl 2014). And in more specific discussions on how to govern climate change, experimentation is increasingly mentioned as a critical part of the way forward (see for instance Hildén et al. 2017). The past decade has seen a steep increase in the adoption and implementation of measures—in the public and private realms, nationally and internationally (Bulkeley et al. 2012; Dubash et al. 2013).

Since agreement at the global level has often proven to be elusive, hopes are increasingly pinned on initiatives at lower jurisdictional levels, on public–private partnerships, on NGOs and other societal actors, and on business-to-business initiatives. Such initiatives are widely seen as experiments (see for instance Hoffmann 2011; Bulkeley et al. 2014; Evans and Karvonen 2014; McGuirk et al. 2015) in a broader governance system that is considerably more polycentric (Jordan et al. 2018). It is said that these experiments are mainly about achieving practical results, but that they nevertheless also “*generate moments in which [past] logics are laid bare for contestation and thus, constitute opportunities for the construction of more progressive outcomes*” (Bulkeley et al. 2014: 1484). Indeed, some suggest that they may have even wider “catalytic” impacts (Hoffmann 2011), as it is hoped that under certain circumstances they create a bandwagon effect.

We identify four topics around which a new, more cross disciplinary debate about experimentation could be organized. The first is crucial and in many ways foundational: the meaning of core concepts. At the moment, there is a *marked lack of conceptual clarity* in the various debates about experimentation. In fact, the term experiment is sometimes used so loosely that anything that deviates from normality—however defined—is assumed to qualify as ‘an experiment’. But, to paraphrase Wildavsky (1979), if everything is an experiment, then maybe nothing is an experiment. To avoid concept stretching, the term should be bounded, and consequences for a more collaborative, interdisciplinary forms of research should be thought through.

The second topic is to do with the *political dynamics surrounding experimentation*. In theory, a wide array of ideas can be tested in policy experiments, but in reality this does not always occur (see Hoffmann 2011). Experiments are not necessarily born equal; they may affect target groups in different ways. Some actors may reap considerable benefits, but others may bear considerable costs (see for instance Castán Broto and Bulkeley 2013). There is a realization that experimentation is not a neutral activity, but since the overall emphasis is on (any) action outside the domain of the state (where *any* action in relation to governing climate change is sometimes perceived as better than international gridlock), the political dynamics of experiments are all too easily assumed away (as already observed by Hoffmann 2011: 156). Paying attention to the objectives behind experiments and/or levels of agreement on the subject of experimentation is thus necessary.

The third topic is *the way experiments are governed* to produce policy-relevant evidence. Experiments are obviously an embodiment of a new idea, but it is worthwhile analyzing who gets to formulate the ideas, who is involved in producing the evidence on their efficacy, which kinds of information should be collected, and which rules of evidence are used. Many politically consequential matters lurk below the surface, for instance, whether one can speak of credible experimental outcomes only when independent scientists are present to evaluate the experiment or not, or whether one can enhance the legitimacy of experiments and their outcomes when their boundary rules are open and many parties can co-experiment.

The fourth and final topic relates to what *experiments produce by way of policy-relevant learning and ultimately policy change.* Ansell and Bartenberger (2016: 70) suggest that the desire to learn is what unites all understandings of experimentation. They suggest that two types of learning dominate: *epistemic learning*, which is about the scientific understanding of the world, and *political learning*, which is about changes in the preferences, goals, and commitments of stakeholders. But in the current debates on experiments in sustainability studies at least, learning from experiments is often taken for granted (McFadgen and Huitema 2017), when in reality experimental evidence may only be one consideration for decision makers. Understanding such effects would probably require a better understanding of the way in which experiments are (strategically) used in the diffusion and evaluation of policy inventions (Jordan and Huitema 2014), although the policy entrepreneurship literature does contain some pointers to the role of “success stories” (see, e.g., Huitema and Meijerink 2009).

Our exploration of these four topics starts in “Experimentation in the social sciences, with a special consideration of the policy sciences”, where we explore the *concept* of experimentation in the social sciences in general and the policy sciences in particular. We show that it is still a niche topic in the policy sciences, but one that nevertheless has considerable lineage. We confirm that several policy-relevant interpretations exist, ranging from those that are quite exclusive (essentially a research *method*) to those that are much inclusive, touching on much broader issues of *governance*. We think that the policy sciences have a great deal to add to and take from debating this topic. Then, we run through the other three topics—*political dynamics*, *governance* and *effects*—and subject them to similar scrutiny, our aim was to identify the main zones of agreement and disagreement and related to that the scope for bringing in the policy sciences. In “Experimentation: new and emerging perspectives”, we discuss what the papers contribute to our understanding of the four topics. We conclude with a number of suggestions on how to develop a new, more coherent and hopefully more cumulative program of work which draws more fully on policy science insights, and which advances our collective understanding of experimentation in social systems.

## Experimentation in the social sciences, with a special consideration of the policy sciences

### Core concepts

The social sciences have a long tradition of reflecting on experimentation, much of it going back to early 20^th^ century thinking on pragmatism. Ansell and Bartenberger (2016: 65) suggest that pragmatist thought considers means and ends to be interrelated. A key concept in pragmatism is “experimentalism”, which is the:

process of iterative adaption to new circumstances and experiences that entails a certain idea of progress and improvement but no teleological endpoint. This perspective leads to an appreciation for historicity and to a conception of growth as a continuous reconstruction of experience.

They subsequently discuss how both Charles Peirce and John Dewey did much to elaborate on the notion of experimentation, albeit from different angles. Residues of their thinking persist in current debates. Peirce elaborated on experimentation as a *method for scientific research* and was one of the first to discuss the role of randomization in experiments (ibid.: 65). This approach includes active interventions or treatments, randomization and statistical analysis aiming at producing valid evidence about cause and effect, moving closer to the Baconian ideal of experimentation (Weiland et al. 2017).

By contrast, Dewey sought to make experimentation a central phenomenon in democracy and ethics, thus emphasizing the need for probing, and trial and error in solving private and public problems. Thus for him, experimentation is seen as an *approach to governance*; in fact, the very essence of experimentation is to try out new approaches in practice. Ansell and Bartenberger (ibid.: 66–67) suggest that two variants of this perspective exist. The first they call “Darwinian experimentalism”, which focuses on systems or ecologies of innovation and emphasizes high levels of diversity so that many diverging approaches are tried out. The second they refer to as “generative experimentation”, which is essentially about trying out one specific innovation and constantly improving upon it on the basis of experience.

Although he obviously affected quite a few other disciplines, Donald T. Campbell is probably the most well-known exponent of experimentation in the policy sciences (see Campbell 1997; Dunn 1997). He considered experiments to be the gold standard for scientific research and saw randomization as the defining feature of experimentation. However, Campbell also developed a broader vision of how experimentation could contribute to better governance. Campbell formulated the Utopian ideal (in his words) of an *experimenting society*. He surmised that it would be characterized by a preference for decentralization and diversity, an inclination towards action rather than inaction, a premium on honest assessment based on transparent data produced in an accountable manner, and a willingness to change theories and values in the face of disconfirming evidence. Subsequently, much of his work was devoted to the methods through which experiments could help advance learning, which for him had to be contrasted with non-reflective and overly ideological approaches to governing. Campbell’s influence can still be felt today. His thinking still influences those who wish to use experiments as *a research method* (see for instance McDermott 2002). Howe (2004: 42) offers a good summary of relevant discussions amongst them, which focus for instance on the question whether experimentation should be seen as a standalone research method or part of an ensemble of methods, whether to accept deviations from a strict experimental designs or not, and whether to allow for qualitative (instead of quantitative) data collection. Campbell’s work has also informed more recent work, for instance on forms of experimentalist governance (see for instance Dorf and Sabel 1998 & Sabel and Zeitlin 2008).

Despite Campbell’s influence, it is fair to say that experimentation has not been the center of attention in the policy sciences. Very few textbooks even index the concept (Anderson 2006; Parsons 1995). The recent edited volume by James et al. (2017) is probably one of the first seek to renew interest in experimentation, in line with what Howe (2004) dubbed mixed-methods experimentalism (seeing experiments as one research method, part of a suite of methods). They focus on experiments as a research method and apply relatively strict definitions when it comes to what counts as an experiment (randomization is considered key), but experimentation is also placed alongside a range of other research methods. As part of their volume, Li and Van Ryzin (ibid.) reviewed the number of experimental studies in “public management journals”^1^ and found that the number of articles describing experimental studies hovered at only 2–4 per annum in the early 1990s, climbing quite slowly to around to reach a peak at 18 per annum in 2015 (see Li and Van Ryzin 2017). Furthermore, it is remarkable how studies in the policy sciences tradition that do refer to experimentation have mostly focused on a limited number of policy fields, notably social policy and education (see for instance Greenberg et al. 2003, who studied 143 experiments in social policy). In almost all such studies, experimentation is approached as a research method and quite a strict (neoclassical) definition of what constitutes an experiment is applied. It is fair to say that this is the dominant conception in the policy sciences.

### Key political dynamics^2^

Policy scientists have pointed out that experimentation—also when applied as a research method—is not a neutral activity; far from it. This does not only concern the situation *surrounding* experiments; politics are also abundant *within* them. This is because the choice of measures to study and the interpretation and presentation of the results often depends on the values and influence of the persons and institutions involved (see, e.g., Guba and Lincoln 1989). Indeed, the work of Brodkin and Kaufman (2000), on experiments in social policy, shows how experiments are infused with political ideas and they suggest that in practice experiments often confirm existing ideas rather than challenge them.

Sanderson (2002: 13–17) studied the British experience with experiments under the New Labour governments in the UK, which were keen to emphasize evidence-based policy making—a context that is in theory was relatively conducive to experimentation. Experiments were meant to provide an evidence basis for policy, but Sanderson found that the ruling party was mostly interested in showcasing their preferred approaches by means of pilots, rather than openly testing lots of ideas. As a correlate of that, experimental settings were often not very representative for ordinary policy making contexts, as extra resources were provided to increase the chances of a successful experiment.

To that, Brodkin and Kaufman (2000) add that the ever changing political context provides challenges and opportunities for those advocating certain new ideas. The ideas guiding particular experiments may or may not stay in vogue for long. This means that by the time experiments start to produce evidence, the political landscape around them may have changed. Thus, experiments may serve as time capsules from a previous era. Interpreting experiments—irrespective of their ideological pedigree—is also an inescapably political process, with various opponents using the experiment as an instrument of advocacy.

### Governing experimentation

Because the dominant conception of experiments in the policy sciences is that they are mainly a research method, proponents of experimentation have paid limited attention to issues of governance, viewing them mostly in quite narrow, methodological terms (e.g., how necessary is a control group, how important is randomization, how relevant is the category of quasi-experiments?) (see for instance Howe 2004). But in the period between the Second World War and the 1970s, there was a fair bit of development in the thinking about the right *scale of experiments*. This was an era in which the role of government in many societies expanded rapidly and the rational planning model was in vogue (Huitema et al. 2009).

At the heart of this thinking was rationalistic policy analysis: decisions should be based on a scientific analysis of the various issues at stake. It was assumed that government led planning should guide societal development and that utilitarian logic (as expressed in cost–benefit analysis) should guide decision making. In the post-war period, the assumption was that governments could and should intervene, where necessary with large-scale experiments in societal systems (see, Van Gunsteren 1976). But in the current era of smaller and leaner government, a pronounced aversion to large-scale experimentation has taken hold (see Bobrow and Dryzek 1987: 142). Instead, notions such as “piecemeal engineering” (Popper 1985 [1944]: 309) and “trial and error learning” (Collingridge 1992) came to be seen as more appropriate guides for policy making. The notion of evidence-based decision making was another symptom of this trend; the onus nowadays is on the government to demonstrate that an intervention is warranted. When such evidence is lacking, the starting assumption is that the government should refrain from action. In other words, experiments become the means to demonstrate the need for policy change, as opposed to the means for effecting change.

Finally, Greenberg et al. (2003: 46) add that the notion of experimentation works best for a situation in which there is a single decision maker with clear goals, a limited set of well-known policy alternatives, and sufficient time to await the outcomes of research. Because we live in times of doubt about the power of big ideas (and solutions) and a world arguably characterized by more “institutional voids” and less ‘big government’ (Hajer 2003), it is not surprising that there has been a turn back to experimentation, only smaller, more exploratory and much more modest in its aspirations.

### The impacts: learning and change

In the 1960s, Harold Lasswell suggested that experiments serve three purposes: improve policy making practices, generate scientific knowledge, and build capacity to implement novel ways of doing policy. For Parsons (1995: 552), all three purposes presume a certain level of learning and a subsequent translation into policy practices. Bobrow and Dryzek (1987) suggested that learning from experimentation needs to be seen in light of a Popperian philosophy of science in which all knowledge is seen as temporary and open to falsification. In this vein, policies are to be regarded as tentative hypotheses—they should be based on the best available theory and should be critically assessed by building up from small scale experiments (hence the aforementioned piecemeal social engineering). The discussion about the outcomes of such tests should be open to anyone who wanted to participate in the debate so that a free interplay of proposals and criticisms can ensue (something that Donald Campbell referred to as a “dialectic of experimental arguments”) (see Bobrow and Dryzek 1987: 140). Knowledge about the outcomes of experiments should certainly not be monopolized by technocrats. Bobrow and Dryzek (ibid.) suggest, however, that these ideals have since lost their appeal, because organizers of experiments are generally less interested in talking to the general public than “in establishing the client–analyst relationships that yield the resources that field experiments require” (ibid.: 140). This is quite a sweeping statement to make, but if correct, the ideal of *democratic* experimentalism (as opposed to small scale, technocratic experimentalism) will probably remain forever unrealized.

There are also several skeptics in the policy sciences when it comes to the learning potential of experiments, understood as a research method. For example, Frank Fischer (1995) has suggested that experiments potentially stifle intellectual progress. This is because, unlike the pragmatists, Fischer beliefs they focus exclusively on means and not goals. Moreover, experimentation can test only one idea at the time, and in any case always absorbs significant time and resources. This echoes Bobrow and Dryzek (1987: 148) who claimed that the class of problems for which experimentation is suitable is probably so small that it is bordering on the non-existent. They say experimentation is appropriate when there “is a well-structured, reasonably static, and highly decomposable problem at hand, with consensus on the criteria to be applied to it”. One does not go as far as labeling climate change a wicked problem to acknowledge that it fails to pass this test.

The policy sciences offer few insights into the way experimental results translate into policy change, although a broader literature of course exists on how policy evaluation influences policy. This literature was surveyed by Mark and Henry (2004), but they made no mention of experiments as a factor explaining policy influence. There is some mention made of the use of experiments by policy entrepreneurs, however. Roberts (1992) claims entrepreneurs use experiments to test the survivability of their innovations. Conversely, John (2017) has suggested that anyone who instigates an experiment qualifies as a policy entrepreneur.

## Experimentation: new and emerging perspectives

The roots of the social science debate on experimentation can be traced back to the pragmatists, some of whom advocated experimentation as a research method, others as an approach to governing. In the policy sciences, the debate about experimentation is largely preoccupied with experimentation as a research method, and quite a few authors have suggested there are many limitations and potential complications. This stands in sharp contrast with the high expectations in the debate on climate governance. Do these challenges simply not exist in these two realms? If not, should we as policy scientists amend our expectations about policy experiments? Or perhaps should those writing about experiments in climate governance heed the many warnings issued policy scientists? In this section, we reflect on these questions by drawing on the main findings of the papers in the rest of this issue. We arrange them under the four topics identified in the first section. So first we focus on *conceptual* discussions and advances, then on insights regarding the political *dynamics* surrounding experiments. Then, we move to insights in the way experiments are *governed* and finally we focus on the *impacts* of policy experiments. Interestingly, none of the contributions to this special issue is concerned with understanding experiments as a research method; all focus on experimentation as an approach to governance. As we will see, this has implications for the analyses and conclusions that follow.

### Conceptual perspectives

In their contribution on the role of experimentation in the way Dutch water managers handle climate change issue, McFadgen and Huitema (2018: X) define a policy experiment as “a temporary, controlled field-trial of a policy-relevant innovation that produces evidence for subsequent policy decisions”. This definition highlights the deductive nature of experimentation, in the sense that the underlying assumption is that experiments should by definition have an underlying (action) theory, which can be proven correct or incorrect. This criterion eliminated most of the experiments on the long list of projects they assembled from self-reporting exercises conducted by Dutch water authorities. In fact, of the 180 projects that policy makers regarded as experiments, only 14 contained an intervention theory. One possible implication of this finding is that the word experiment is used differently in practice, i.e., denoting an intention to do something novel, which follows an inductive logic.

In their study of the way experiments can contribute to radical societal transformations, Bernstein and Hoffmann (2018) also embrace this more inductive logic. They conclude that various conceptualizations of experiments exist, but that all “share the notion that something new is being tried out—there is a conscious intervention that differs from the status quo” (Ibid. X). They cite Abbott (2017) who suggests that experiments can be formal or informal depending on the level of conscious experimentation and control over the process. Formal experiments denote analogs of controlled laboratory experiments and informal experiments refer to “a more metaphorical understanding that views climate governance experiments as novel attempts at governing climate by non-traditional global actors […]” (Bernstein and Hoffmann 2018: X). They place themselves at the informal end of the spectrum, by focusing on the activities of subnational actors (states, provinces, cities, but also civil society and company initiatives) in the context of global discussions about climate change. They suggest that when the global discussion gridlocked, subnational actors took over.

Bernstein and Hoffmann (2018) present a potentially very interesting conceptual innovation when they try to develop a simple model of the way experiments alter reality. The dependent variable in their model is the effect that experiments have on lock-ins in the way that economies operate, principally those increasing their dependence on fossil fuels. Sharing an insistence on the political character of experiments they are particularly interested in: (1) the politics that experiments produce; and (2) the subsequent pathways for change that they create, hopefully in the direction of “decarbonization”. Regarding topic (1), they propose that experiments affect what is considered normal (“normalization” which is about the framing and reframing of what is appropriate action), how capacities are directed (in a material, institutional and cognitive sense), and which coalitions develop (compare some of the forms of learning, mentioned by McFadgen and Huitema (2017)). They explicitly indicate that each act to affect framing, capacities, or coalitions is bound to result in counter action. Regarding Topic 2, they suggest that, depending on the nature of the politics that emerge, experiments can reinforce existing lock-ins, help improve such lock-ins, or they can lead to a breaking up of the lock-in and help decarbonize the economy.

One tantalizing theoretical advantage of thinking about lock-ins is that an experimental intervention in one element of an interlocking system may create disturbance (and thereby potentially effects) in other parts. To describe these effects, Bernstein and Hoffmann (2018) introduce the terms scaling and entrenchment. Scaling has to do with the way in which a successful experiment leads to more experiments, larger-scale experiments, or experiments on other jurisdictions. Entrenchment relates to the stickiness of innovations introduced by experiments—some new policies may immediately get locked-in (as if they had always been there), some policies have rising benefits over time, and new populations may join the new policy.

Voß and Simons (2018), in their study of the way emissions trading become a credible and legitimate policy option, make an important conceptual claim about experiments. They go beyond Lasswell’s notion that experiments affect reality in part by capacity building. They explore the *reality shaping nature of experiments*; if and when an intervention is deemed successful, the manipulation in question will form the basis of further attempts to model and reshape reality. They suggest experiments can perform two important roles, which they refer to as *scientific reality making* and *political reality making*. Both forms involve similar acts, such as demonstrating a condition, justifying claims, and establishing order. But there are strong differences in what is at stake in the lab and in the field, i.e., epistemic versus political authority. The type of evidence considered is different too, as are the obligations considered.

### The political dynamics of experimentation

Voß and Simons (2018) side with policy scientists when they contend that experiments are intrinsically political in nature. This is because neutral observation is impossible and because the starting premises of an experiment are often highly consequential. They conceptualize experiments as entities operating at the interface between science and policy. They contend that these two fields are more interwoven than is usually acknowledged. One of their most significant contributions is to show how experiments play a role, which is not so much by means of a one-off attempt to produce evidence, but rather in a longer and convoluted process which may actually involve multiple experiments (but also other ways of gathering evidence). By dissecting the long and arduous path that the instrument of emissions trading had to accomplish, Voß and Simons show how it involved no less than five experiments. *Lab experiments* contributed to the epistemic authority of the idea and *field experiments* contributed to the formation of political support. They also underline the importance of time: experimentation with emissions trading evolved over many decades, thus aligning with the idea that experiments may serve an indirect (or enlightenment) function over the very long term (Weiss 1977).

The contribution by Rocle and Salles (2017) also demonstrates the political sensitivities surrounding experiments. In their analysis of what was essentially a *thought experiment* with planned retreat from the eroding coastline in the Aquitaine region of Southwest France, they show how much the initial framing of the experiments mattered. The French national government was considering planned retreat, but encountered much local resistance to the idea. Volunteer communities were asked to come forward and receive money for a participatory process that would actively consider retreat through a scenario based approach combined with back casting. Leading politicians in one of the few municipalities to come forward and join the experiment emphasized that the choice to retreat should be a local one. It would not be forced upon them—hence the request for “pioneers, not Guinea pigs” (ibid.: X). The central government in turn was keen to emphasize the fact that the experiment was a shared responsibility, with the national government working with local governments to experiment with new ideas about coastal management.

### The governance of experiments

McFadgen and Huitema‘s contribution focuses on the way experiments can be governed. They suggest that the *institutional design choices* that initiator of experiments must take go beyond the choice for an intervention, randomization and presence of a control group. Based on Ostrom’s (2005) Institutional Analysis and Development framework, they suggest that initiators of experiments need to make several choices concerning the type of information that is regarded or ignored, the authority to make decisions on the experiment, the costs and benefits associated with the experiment, etc. They present a set of ideal type experiments, which can serve as a tool for the interpretation of real life experiments. Leaning on Pielke (2007), they suggest that design of experiments can rely mainly on technocracy, on advocacy, or on brokerage at the boundary between science and politics (the key point being that experiments are not intrinsically technocratic as is often assumed). Their typology is able to detect subtle differences between 14 experiments in Dutch climate adaptation, which suggests that it may have analytical value elsewhere too.

### What changes? Experiments, learning and change

Bernstein and Hoffmann (2018), in their quest to understand the disruptive force of experiments, provide a test run of their model on a few real world examples. To that end, they apply it to three case studies, one that reinforced existing lock-ins (Colorado’s New Energy Economy initiative), one that unlocked carbon lock-ins (the UK government-sponsored Carbon Trust), and one that has the potential to decarbonize (Copenhagen’s climate policy). They find that in Copenhagen it was normalization that contributed to the scaling and entrenchment of the notion of carbon neutrality. This occurred by consistently framing climate action as contributing to the improvement of life in the city—through the slogan the Good Life = Sustainable Life and a Green City = Economic Growth. They warn, however, that the “potential or trajectory [of experiments] generally cannot be calculated a priori” (Bernstein and Hoffmann 2018: X). But they do suggest that their framework sheds light on the political dimension, as it *“provides a way to identify and track the political forces and mechanisms through which experiments impact targets of intervention and make (or fail to make) broader connections”* (Bernstein and Hoffmann 2018: X).

Rocle and Salles (2018) confirm some of the bold predictions made by Voß and Simons (2018) about the reality creating effect of experiments. They show that simply discussing planned retreat made the notion more acceptable and at least changed the local political discourse. They also show how policy entrepreneurs—in their case, a collaboration of local authorities—played an active role in setting up the experiment, in gaining local acceptance by framing it in a positive light, by navigating political dynamics emanating from local elections and in connecting across jurisdictional levels. The fact that the committee monitoring the experiment included two national parliamentarians was not a coincidence; in fact it actively facilitated the transfer of lessons—namely adapting national legislation to make planned retreat a viable option. Because this was always the aim of the national government, Rocle and Salles hesitate to speak of social learning beyond the very local level. In effect they side with Castán Broto and Bulkeley (2013) who have suggested that experiments are one way through which visions of the future are rendered practical and hence governable (compare Voß and Simons 2018). It the typology of McFadgen and Huitema (2017, 2018), the French coastal experiment was very much an advocacy experiment, with relatively high degrees of openness in terms of which kind of actors could participate. But there was less openness in terms of the expected outcomes: the experiment was much more a means to gauge and ultimately govern local responses to rising sea levels.

McFadgen and Huitema (2018) are interested in the conceptual utilization of experimental results; that is, how the experiments influence the mindsets of policy makers (and especially elected politicians). Following Weiss (1977) they suggest that experiments are likely to play a role in the gradual sedimentation of ideas in policy making, yet they also suggest that measuring such effects over years or even decades is very difficult. Instead they propose three proxy indicators that gauge the short-term reaction of policy makers to experiments by assessing the degree to which policy makers considered the results salient, credible and legitimate (see Cash et al. 2003). In addition, McFadgen and Huitema hypothesize that the way an experiment scores on the three criteria vary under the influence of the institutional design. On the basis of data from 164 online surveys targeting policy makers who know about experiments (many of them did not), they tested several explicit hypotheses, for instance that “technocratic experiments” score higher on credibility than the other two types (“advocacy” and “boundary” experiments, respectively). Statistical analysis revealed that institutional design had a significant effect, but surprisingly some intuitively plausible hypotheses were rejected. For example, technocratic experiments, despite their emphasis on science and scientific impartiality, did not score higher for credibility than the other two types. In addition, advocacy experiments scored higher for legitimacy; this was surprising because of the one-sided and somewhat closed nature of their institutional setting. They assume this is to do with the relatively low level of conflict over policy goals in the Dutch water management community, and speculate the scores might have been lower in more conflictual settings.

## Conclusions and new directions

The expectations surrounding the experimentalist turn are sky high, but we have suggested that four topics deserve much more attention. To obtain more insight, we took proceeded via two steps: we gave a short overview of the discussion about experimentation in different literatures including the policy sciences and then we discussed the various novel contributions contained in this special issue. In this final section, we draw conclusions on the possibilities for mutual learning across the disciplines and identify directions for further research.

### Towards common concepts?

In terms of core *concepts* (our first topic), we have shown how experimentation has long been debated across the social sciences. In pragmatist thought, two different ways of looking at experiments can be discerned: experiments as a research method and experiments as an approach to governing. In the discussion on experimentation in the policy sciences, the emphasis has overwhelmingly been on experiments as a research method (i.e., specifically one that requires randomization, control groups, etc.). By contrast, most authors writing about sustainability and in particular climate governance, see experimentation as an approach to governance.

On reflection, some of the confusion that has arisen is probably down to the fact that scholars are talking past one another, with some prominent policy scientists claiming that the term experiment has been “somewhat inaccurately” defined in the climate governance literature (see Dryzek 2017: 789). It is probably not that helpful to count every form of policy making and/or governance as ‘an experiment’. In this debate, McFadgen and Huitema (2018) helpfully identify a middle ground which a shared understanding could be achieved at the intersection between fields. They suggest that at minimum two basic conditions should be satisfied before something is labeled as an experiment: there should be an intervention theory with explicit assumptions (or hypotheses) which are tested and there should be some novelty. Hence, they write: “the act of experimentation should be explicit: without appraisal of the intervention’s effects, there is only demonstration of a new initiative, and without innovation, only established ideas are being evaluated” (McFadgen and Huitema 2018: X). Admittedly, their suggestion rules out many aspects of experimentation that are quite common in the climate governance literature (e.g., the assumption that an experiment is any initiative that occurs outside the international regime for instance). But it is inclusive enough to include experiments as a research method and experiments as a means of governance.

Applying such a definition could have important implications for the policy sciences. These go beyond the obvious observation that a shared understanding would allow us to study a number of specific cases and thus more rapidly enhance our empirical understanding of experimentation. Beyond the obvious, there may also be other consequences. Regarding the very practical ones, Voß and Simons (2018) demonstrate that the inclusion of both types can have added value since practitioners would be able to apply both types in one decision process—something they would have missed if they had applied a very strict definition. On a more profound level, taking a broader definition of experiments could create a means to connect to the pragmatist philosophy of Dewey and others.

### What are the key political dynamics?

This leads logically to our next conclusion, which relates to the political *dynamics* surrounding experiments (our second topic). We have argued that in the debate on climate governance experiments political issues have largely been downplayed because experiments are seen as a way to *evade the politics* bedevilling inter-state diplomacy. Policy scientists have repeatedly sought to play up this aspect. For instance, many discussions of Campbell’s ideas have revolved around the compatibility between taking risks in an experimenting society and basic democratic values (see Dunn 1997). Would the role of ideology not be diminished in an experimenting society? Do scientists crowd out elected politicians in the decision making process? How does society avoid the selective use of scientific evidence? How will ordinary citizens be involved in decision making? Note here that speaking of the politics *surrounding* experiments (as we did ourselves at the outset of this article) is constraining, because such language may imply that the experiment itself is free from politics, whereas for many policy scientists politics are an *intrinsic* part of experiments. Unlike many publications on experimentation in climate governance so far, the contributions to this special issue directly address the political nature of experiments. The notion that experiments create new realities and affect discourses comes out very strongly in all the contributions.

This special issue also advances our understanding of the interaction between science and policy. In many literatures, this is largely seen as a cognitive process of enlightenment—of unsettling and breaking up existing power arrangements. The model that Bernstein and Hoffmann (2018) offer is potentially innovative and could be considered for wider use. They suggest that experiments can change expectations about what is normal, build capacities, and affect coalition formation. They also suggest that experiments should be analyzed over time, asking whether they have actually broken up existing situations, have actually unwittingly perfected them, or have left them as they originally were.

### The governance of experiments: understanding the stakes

We propose that the current stringent way of studying experiments in the policy sciences (i.e., seeing them as a research method) has limited the ability of policy sciences to offer reflections on the *governance* of experiments (our third topic). Indeed, when experiments are viewed purely as a research method, the whole issue of governance becomes narrowly framed: e.g., the hypotheses to be tested; the characterization of the control situation (or group); the comparison of treatment vs non-treatment options, etc. However, under a broader understanding, the way the governance of experiments is set up, does become an issue as suggested by McFadgen and Huitema (2018).

We think that their idea that experiments could be designed in various ways (e.g., in a technocratic way, but also as a boundary object, or as a tool for advocacy) potentially has important implications. At a conceptual level, it means that experiments need no longer be exclusively associated with technocracy. This is significant because possible tensions between the proper functioning of democracy and experimentation surface in almost every publication on experimentalism or experimentalist governance. Such tensions will not completely disappear if experiments are understood as boundary objects or advocacy tools. In fact new tensions could well appear—for instance when the capacity to experiment, or access to experimental results, is distributed unequally in societies. However, the impression that experimentalist governance essentially means rule by experts would be taken away. The fact that experiments can also be undertaken in a non-technocratic way would actually embolden those who argue that experimentalist governance should operate as a (democratic form of) “directly deliberate polyarchy” (Sabel and Zeitlin 2008: 276).

At the empirical level, this insight offers the potential to acquire a more detailed picture of what it is that experimenters do in practice—the stock in trade of many policy scientists. In fact, accepting the idea that an experiment can sometimes be a tool of advocacy or a boundary object would allow us to bring the politics back into experimentation. It would also help us form us a better understanding of the potential impacts. Here, we draw on the insights proffered by Voß and Simons (2018), who emphasize the performative aspects of experiments and the craft that goes along with this. As noted, they suggest that sometimes experiments are intended for political reality making, sometimes for scientific reality making.

### What actually changes? The impacts of experiments

This brings us to the matter of *impacts* that experiments have, whether through learning or policy change (our fourth topic). Here too, the idea that experiments can be governed in multiple ways has implications for the way we study them, because it draws attention to their internal dynamics. If not all experiments have (by definition) to be technocratic, then it becomes important to study the possibilities for learning from experiments that go beyond simple scientific conclusion drawing. Indeed, if experiments are treated as boundary objects (see McFadgen and Huitema 2018), then it encourages analysts to study their effects on the norms that their organizers bring to the problem—something that has so far largely escaped the attention of policy scientists. The question of what happens to the findings of experiments in the broader policy setting has obviously not escaped attention. In fact it is well known that politicians can ignore the results that they cherry pick from the conclusions, etc. Interestingly, insights into how experiments affect learning and policy change have yet to be included in most of the leading models of the policy process. This feels like an omission after that more policy scientists should start to address.

Several papers in this special issue also underline the importance of the time dimension. McFadgen and Huitema (2018) propose that the three criteria of salience, credibility and legitimacy may serve as short-term indicators for the impact that experiments may eventually have in policy circles. They do so on the basis of an assumption that the outcomes of experiments with higher scores for these criteria have a higher chance of being taken on-board by policy makers. However, they do not test this assumption, but rather flag it as a matter deserving further research. Voß and Simons (2018) also take a very long perspective on emissions trading. They show the added value of paying attention to both experiments as research method (what they call lab experiments) and experiments as an approach to governance (which they refer to as field experiments). Applying the thinking of Bernstein and Hoffmann, one can easily see how the experiments with emissions trading indeed led to capacity building, to normalization, to coalition building, and eventually to anchoring of this instrument.

In summary, conceptual precision is needed, especially in the realm of what counts as an experiment. The political dynamics surrounding and within experiments should also receive more attention because experiments are not neutral endeavors and their use potentially invokes difficult questions about the proper relation between science and policy. In addition, experiments can be governed in multiple ways: many important decisions have to be made about the types of information that count, which participants can become involved; and the way evidence is established, etc. The consequences of such “design questions” need to be understood if, as seems to be the case, more governors wish to experiment. Finally, it remains unclear which factors determine whether certain experiments lead to learning and policy change. The contributions to this special issue offer important suggestions for taking forward the debate on these topics. Whether or not we need an experimentalist turn in the policy sciences is very much an open question. The prospects seem tantalizing as insights on the way experiments function is potentially very relevant (for instance) for an update of our theories on the policy process, on policy learning, for insights on the interactions between formal and more informal forms of policy making, and for theories on the functioning of the science-policy interface. But our argument is that such a turn will probably not add up to as much if we keep treating experiments as a research method, as opposed to a broader approach to governing.

## Footnotes

1 This included the Policy Studies Journal, Policy Studies, and the journals Science and Public Policy and Governance, but not this journal.

2 Elements of this and the next subsection draw on Huitema et al. 2009.
